# AAV2-Mediated Combined Subretinal Delivery of IFN-α and IL-4 Reduces the Severity of Experimental Autoimmune Uveoretinitis

**DOI:** 10.1371/journal.pone.0037995

**Published:** 2012-06-07

**Authors:** Lichun Tian, Bo Lei, Ju Shao, Lin Wei, Aize Kijlstra, Peizeng Yang

**Affiliations:** 1 The First Affiliated Hospital of Chongqing Medical University, Chongqing Key Laboratory of Ophthalmology and Chongqing Eye Institute, Chongqing, People’s Republic of China; 2 Guangzhou Institute of Respiratory Diseases, State Key Laboratory of Respiratory Diseases, The First Affiliated Hospital, Guangzhou Medical University, Guangzhou, People’s Republic of China; 3 Eye Research Institute Maastricht, Department of Ophthalmology, University Hospital Maastricht, Maastricht, The Netherlands; University of Florida, United States of America

## Abstract

We previously showed that adeno-associated virus 2 (AAV2) mediated subretinal delivery of human interferon-alpha (IFN-α) could effectively inhibit experimental autoimmune uveoretinitis (EAU). In this study we investigated whether subretinal injection of both AVV2.IFN-α and AAV2.IL-4 had a stronger inhibition on EAU activity. B10RIII mice were subretinally injected with AAV2.IFN-α alone (1.5×10^7^ vg), AAV2.IL-4 alone (3.55×10^7^ vg), and AAV2.IFN-α combined with AAV2.IL-4. PBS, AAV2 vector encoding green fluorescent protein (AAV2.GFP) (5×10^7^ vg) was subretinally injected as a control. IFN-α and IL-4 were effectively expressed in the eyes from three weeks to three months following subretinal injection of AAV2 vectors either alone or following combined administration and significantly attenuated EAU activity clinically and histopathologically. AAV2.IL-4 showed a better therapeutic effect as compared to AAV2.IFN-α. The combination of AAV2.IL-4 and AAV2.IFN-α was not significantly different as compared to AAV2.IL-4 alone. There was no difference concerning DTH (delayed-type hypersensitivity) reaction, lymphocyte proliferation and IL-17 production among the investigated treatment groups, suggesting that local retinal gene delivery did not affect the systemic immune response.

## Introduction

Uveitis is a common eye disease [1] and is one of the major causes of visual handicap within the working population worldwide [2], often with an autoimmune cause [3]. Clinical features include inflammation of the choroid and retina with cellular infiltration and macular edema [4]. Treatment of uveitis commonly involves the use of corticosteroids and other immunosuppressive agents [5]. However, long-term application of these drugs frequently leads to adverse side effects systemically and locally. Furthermore, not all uveitis patients appear to respond to immunosuppressive treatment.

IFN-α has been shown to have immunoregulatory and immunosuppressive effects, and has been shown to be beneficial in the treatment of patients with uveitis [6], [7]. In addition, Th1 cells have been shown to be responsible for the development of uveitis and endogenous Th2 cells have been assigned a protective role. In this regard, IL-4, a Th2-secreted anti-inflammatory cytokine, may help to prevent the development of Th1 related autoimmune diseases. Earlier studies have shown that IL-4 was effective in the treatment of NOD mice, a spontaneous model of autoimmune type 1 diabetes [8]. It was also effective in the treatment of experimental autoimmune encephalomyelitis (EAE), a well known model for multiple sclerosis [9].

Currently, gene therapy has achieved remarkable success in human and animal models in various retinal diseases [10], [11], [12], [13] and some studies are now in the clinical trial stage [14].

Experimental autoimmune uveoretinitis (EAU), an animal model that shares many features with human uveitic disorders such as Behcet’s disease [15], can be induced in susceptible animals such as the B10.RIII mouse strain by immunization with retinal specific antigens that are often recognized immunologically by lymphocytes of human uveitis patients including interphotoreceptor retinoid binding protein (IRBP) or its immunodominant epitopes. Because of the clinical and pathological features in common with human uveitis, EAU induced by IRBP in B10.RIII mice is considered as a useful tool to explore new therapeutic strategies. In the past several years, a few studies on AAV-mediated gene therapy have been attempted in the EAU model [16], [17]. In a recent study we developed a recombinant AAV2 vector containing the human IFN-α gene and revealed that subretinal injection of AAV2 vector harboring the IFN-α gene showed a significant therapeutic effect on the development of EAU [18].

In the current study, we examined if subretinal administration of IFN-α combined with another immunoregulatory cytokine, IL-4, would be more effective in the treatment of EAU in B10RIII mice and if AAV-mediated transgene subretinal delivery had an effect on the systemic IRBP-specific immune responses following EAU induction.

## Materials and Methods

### Ethics Statement

This study was carried out according to the ARVO Statement for the Use of Animals in Ophthalmic and Vision Research. The study was specifically approved by the Ethics Committee of the First Affiliated Hospital of Chongqing Medical University, Chongqing, China (Permit Number: 2009-201009). All surgery was performed under anesthesia, and all efforts were made to minimize animal suffering.

### Animals and Reagents

B10RIII mice were purchased from Jackson Laboratory (Bar Harbor, ME). All animals were housed under standard (specific pathogen free) conditions. Human interphotoreceptor retinoid binding protein peptide spanning amino acid residues 161–180 (IRBP_161–180_, SGIPYIISYLHPGNTILHVD) was synthesized by Shanghai Sangon Biological Engineering Technology & Services Ltd. Co. Complete Freund’s adjuvant (CFA) containing 1.0 mg/ml mycobacterium tuberculosis (H37RA, ATCC 25177) was obtained from Sigma-Aldrich (St. Louis, MO).

### Vectors

The recombinant adeno-associated virus vector harboring the human interferon alpha 2a gene (AAV2.IFN-α) or mouse interleukin-4 gene (AAV2.IL-4) was prepared according to previously mentioned methods [18] as follows. Total mRNAs were extracted from human PBMCs and mouse splenocytes respectively and first-strand cDNA was synthesized with the Superscript III Reverse Transcriptase system (Invitrogen, Carlsbad, CA, USA). The coding sequence of human interferon alpha 2a was obtained from GenBank database (http://www.ncbi.nlm.nih.gov/genbank/, GenBank accession number BC074936) and the specific primers were designed (forward, 5′ GGGGTACCATGGCCTTGACCTTTGCTTT 3′ and reverse, 5′ CTGTCGACTCATTCCTTACTTCTTAAACTTT 3′) to amplify the human IFN-α coding sequence. The mouse interleukin-4 coding sequence was obtained from GenBank database (http://www.ncbi.nlm.nih.gov/genbank/, GenBank accession number NM_021283) and was PCR-amplified with the specific primers (forward, 5′ GGGGGTACCATGGGTCTCAACCCCCAGC 3′ and reverse, 5′ TCTGTCGACCTACGAGTAATCCATTTGCATG 3′). The PCR product was inserted into pMD 18-T Vector (TaKaRa, Japan). After DNA sequencing verification, the IFN-α or IL-4 coding sequence was obtained by digestion with *Kpn*I and *Sal*I, and subcloned into an AAV2 expression plasmid backbone between the sites of *Kpn*I and *Sal*I. Recombinant AAV vector containing the IFN-α or IL-4 gene was prepared by using the triple transfection procedure of 293 cells, followed by CsCl density gradients (Vector Gene Technology Company Ltd (Beijing, China)).

### Subretinal Injection

0.5 µl of AAV vector was injected subretinally into the right eye of each B10RIII mouse, leaving the other eye as an internal control. Subretinal injection was performed as previously described [18], [19] and all procedures were operated under sterile conditions.

### Assay of Interferon-α and Interleukin-4 in Injected Mice

Mice were sacrificed at various time points after subretinal injection of AAV vectors. The undiluted serum was collected for immunoassay. For ocular fluid samples, AAV vector injected eyes and contralateral eyes were enucleated and ocular fluid samples were prepared as previously described [18]. All procedures were conducted on ice. IFN-α and IL-4 concentrations were determined using commercially available ELISA kits according to the manufacturer’s directions (PBL Interferon Source, USA). The detection limits were 12.5 pg/ml and 15 pg/ml for IFN-α and IL-4, respectively.

**Figure 1:**
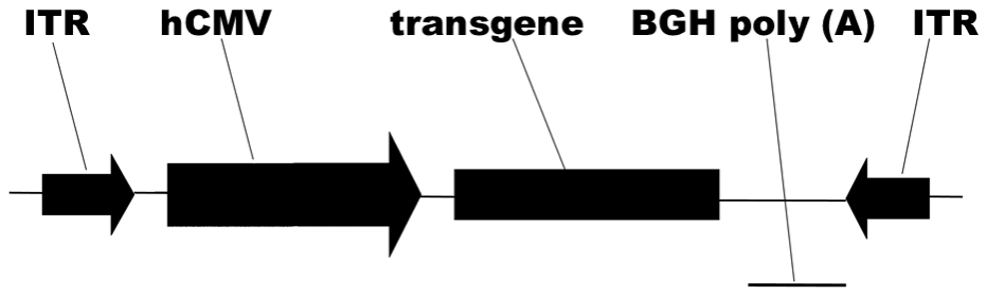
Scheme of the AAV2 vector construct. The transgene is under the control of a CMV promoter and followed by BGH poly (A). The expression cassette is flanked by ITRs. CMV promoter, human cytomegalovirus immediate early promoter; hIFN-α, human interferon-alpha; BGH poly (A), BGH poly-adenylation signal; ITR, AAV2 inverted terminal repeats.

### Induction and Clinical Assessment of EAU

Mice were immunized subcutaneously at the base of the tail and both thighs with 50 µg human IRBP_161–180_ peptide in 100 µl PBS, emulsified 1∶1 v/v in complete Freund’s adjuvant (CFA) supplemented with 1.0 mg/ml *Mycobacterium tuberculosis* strain (MTB). A total of 200 µl emulsion was given for one mouse. EAU activity was examined clinically by slit lamp microscopy from day 8 to 21 after immunization. The clinical severity of ocular inflammation was assessed by two independent observers in a masked manner, and scored on a scale of 0–5 in half-point increments, according to five separate criteria described previously [18].

### Histopathology

Eyes were enucleated on day 14 following IRBP immunization and were fixed in 4% buffered formaldehyde for 1 hour at room temperature. Tissues were embedded in paraffin. Serial 4–6 µm sections were cut through the papillary-optic nerve axis and stained by haematoxylin and eosin. At least four sections of each eye cut at different levels were prepared and evaluated histologically. The intensity of EAU was graded in a masked fashion on a scale of 0 to 4, as described earlier [20]: (0) no change; (0.5) mild inflammatory cell infiltration, no damage; (1) infiltration, retinal folds and focal retinal detachments, few small granulomas in choroid and retina, perivascularitis; (2) moderate infiltration, retinal folds and detachments, focal photoreceptor cell damage, small- to medium-sized granulomas, perivasculatis and vasculatis; (3) medium to heavy infiltration, extensive retinal folding with detachment, moderate photoreceptor cell damage, medium-sized granulomatous lesions, subretinal neovascularization; (4) heavy infiltration, diffuse retinal detachment with serous exudates and subretinal bleeding, extensive photoreceptor cell damage, large granulomatous lesion and subretinal neovascularization.

### Delayed-type Hypersensitity (DTH)

Delayed-type hypersensitity (DTH) was assessed on day 19 after immunization with IRBP_161–180_ peptide. 10 µg of IRBP_161–180_ peptide in 10 µl PBS was injected into the right pinna of each mouse, the left pinna was injected with PBS as a control. Ear thickness was measured 48 hours after IRBP_161–180_ peptide challenge. The specific response was calculated as the difference between ear thickness before and after the IRBP_161–180_ peptide injection.

**Figure 2:**
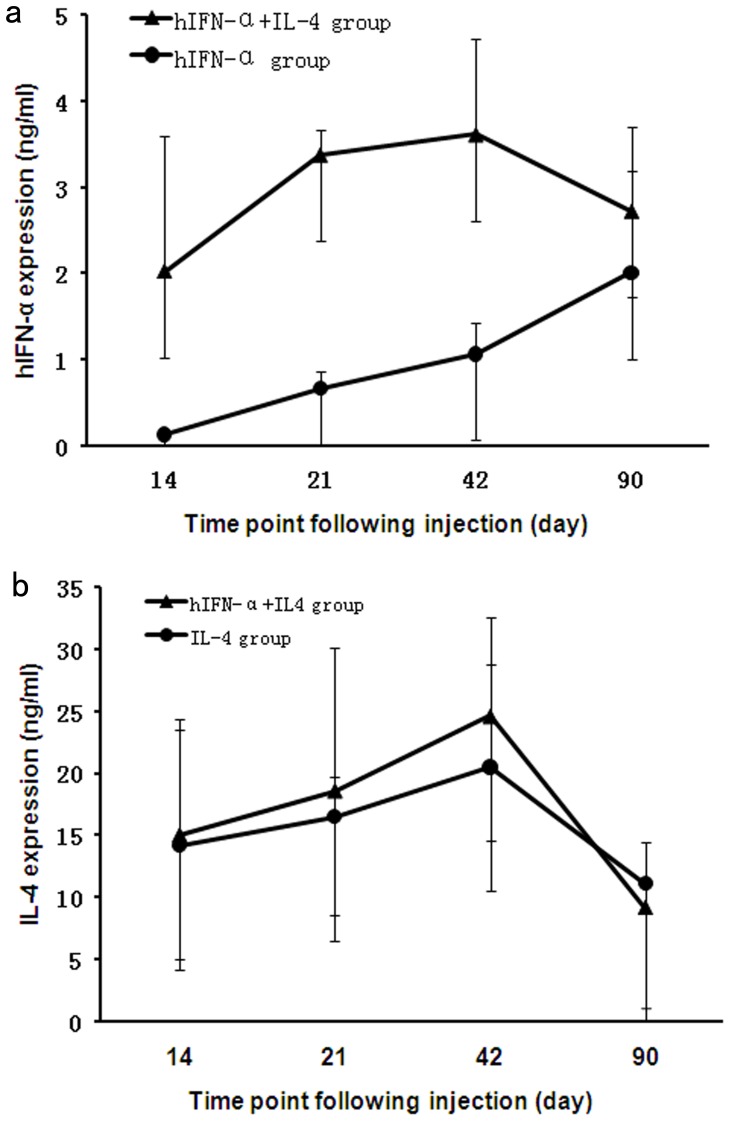
The expression of transgenes following subretinal injection . In the AAV2.IFN-α injected eyes, the level of IFN-α increases from 14 days (the first time point tested) to three months after injection. For the eyes receiving AAV2.IFN-α combined with AAV2.IL-4 injection, IFN-α level reaches a peak on day 42 and remains at a moderate level until three months after injection (**a**). IL-4 expression is similar in eyes receiving an injection of AAV2.IL-4 alone as compared to eyes receiving AAV2.IL-4 combined with AAV2.IFN-α (**b**). Results are expressed as the mean±standard deviation.

**Figure 3:**
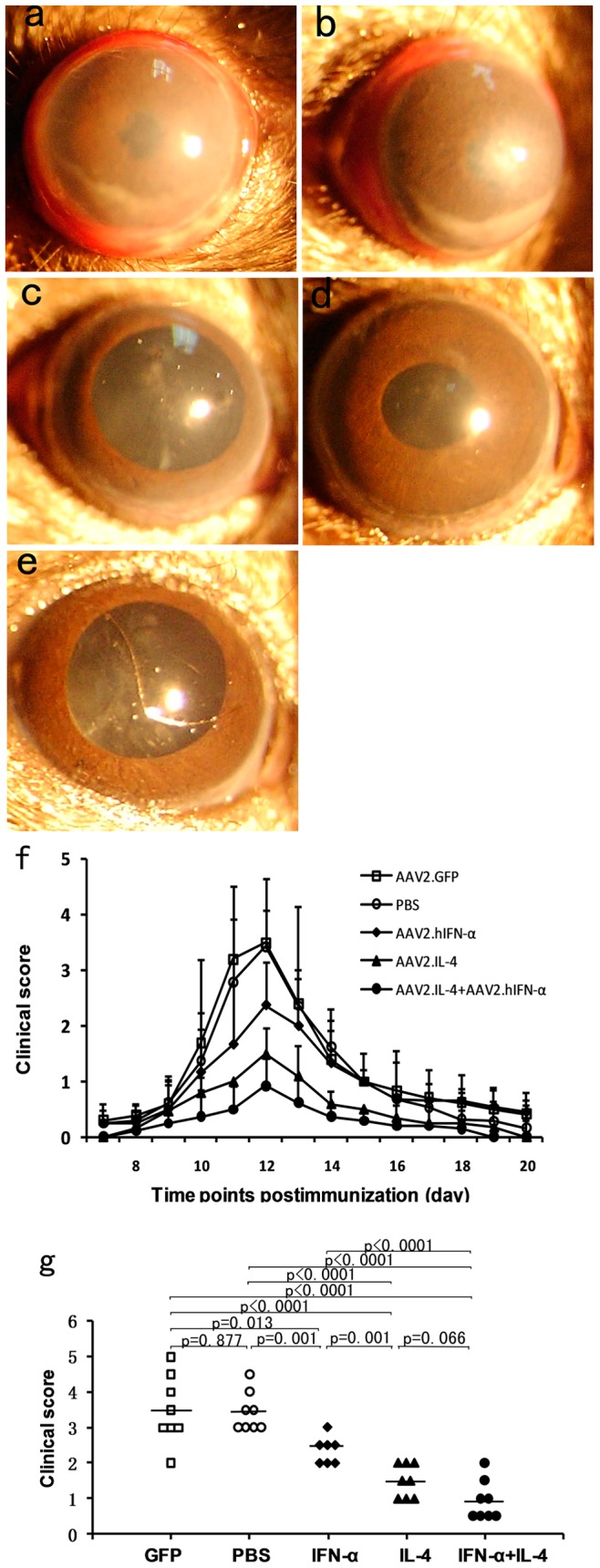
Clinical evaluation of EAU activity. EAU was induced in groups of mice receiving subretinal vector or PBS injection. Severe uveitis is observed in the PBS (**a**) and AAV2.GFP injected eyes (**b**) as compared to the AAV vectors treated eyes (**c–e**). Three AAV vector treated groups show significantly attenuated EAU over time as compared with controls (**f**). Data are presented as mean±standard deviation. The clinical score shows that compared with controls, the AAV2.IFN-α treated group (p = 0.019, Mann-Whitney U test), the AAV2.IL-4 treated group (p<0.0001) and the combined treated group (p<0.0001) developed a significantly reduced EAU (**g**). Each point represents an individual eye. The average scores of each group are denoted by the horizontal bars.

### IRBP-specific Lymphocyte Responses

The spleen and draining lymph nodes were removed from immunized mice on day 21. A single cell suspension was prepared by mechanical disruption and followed by a passage through a sterile stainless steel screen. For proliferation and cytokine assay, cells (2×10^6^ cells/ml) were cultured in triplicate with RPMI 1640 medium (Gibco, Grand Island, NY, USA) containing 2 mM L-glutamine, 5×10^−5^ M 2-ME, 0.1 mM nonessential amino acids, 1 mM sodium pyruvate and 10% FBS in the presence of 10 µg/ml IRBP_161–180_, 1 µg/ml Concanavalin A (Sigma) or medium alone for 72 hours. Proliferation was detected by a modified MTT assay using a cell counting kit (Cell Counting Kit-8; Sigma) as described previously [21]. IL-17 concentration in the supernatants was measured using a commercially available ELISA kit according to the manufacturer’s directions (R&D System, Minneapolis, MN) with a detection limit of 15 pg/ml.

### Statistical Analysis

Severity of EAU was performed by the Kruskal-Wallis analysis followed by the Mann-Whitney U test with Bonferroni correction. DTH and IRBP-specific immune responses were analyzed using ANOVA. P<0.05 was considered to be significantly different. Data are expressed as mean±standard deviation (SD). All experiments were repeated at least twice.

**Figure 4:**
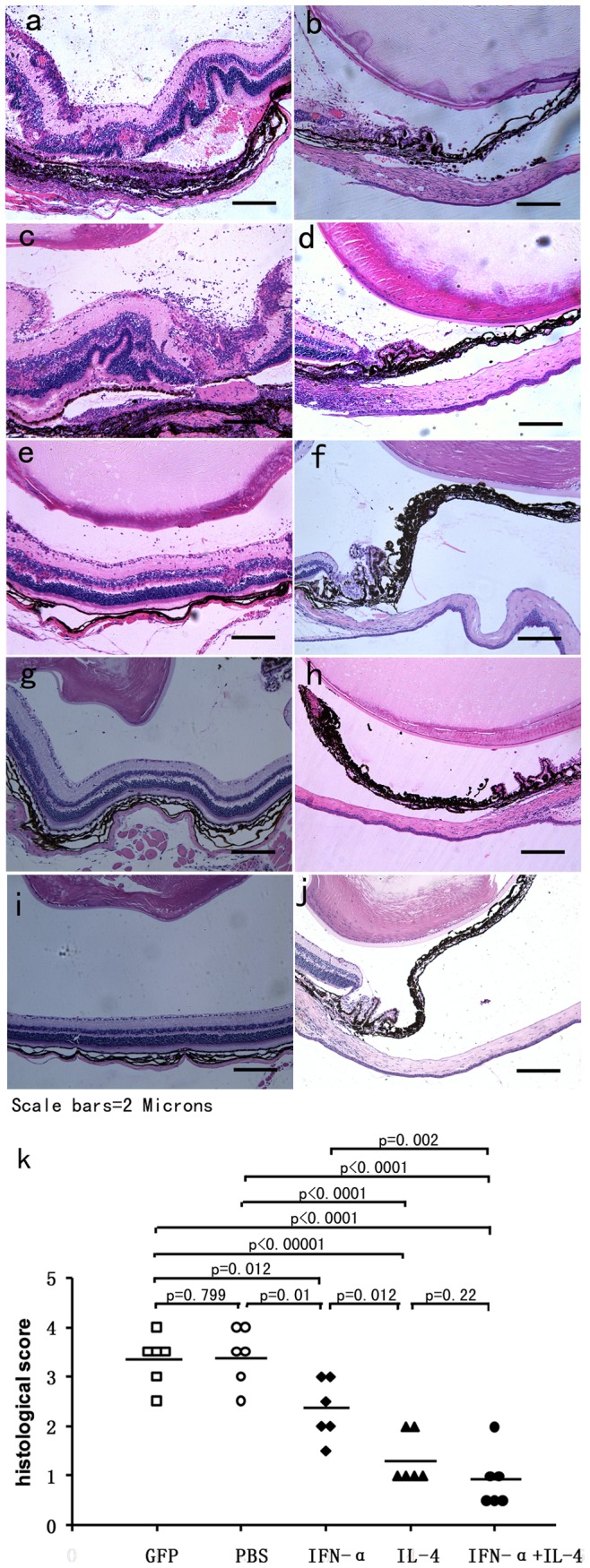
Histological examinations on day 14 of EAU. Images of histological analysis show severe intraocular inflammation in PBS (**a,b**) and AAV2.GFP injected eyes (**c,d**) compared with AAV2.IFN-α treated (**e,f**), AAV2.IL-4 treated (**g,h**), and AAV2.IFN-α combined with AAV2.IL-4 treated eyes (**i,j**). (haematoxylin eosin staining, original magnification×100). EAU was significantly reduced in AAV2.IL-4 treated group, AAV2.IL-4 combined with AAV2.IFN-α treated group as compared with controls (**k**) (p<0.0001, Mann-Whitney U test). The AAV2.IFN-α treated group also shows a significantly decreased uveitis (p = 0.005). Each point is the score of an individual eye. The mean scores of each group are denoted by the horizontal bars.

**Figure 5:**
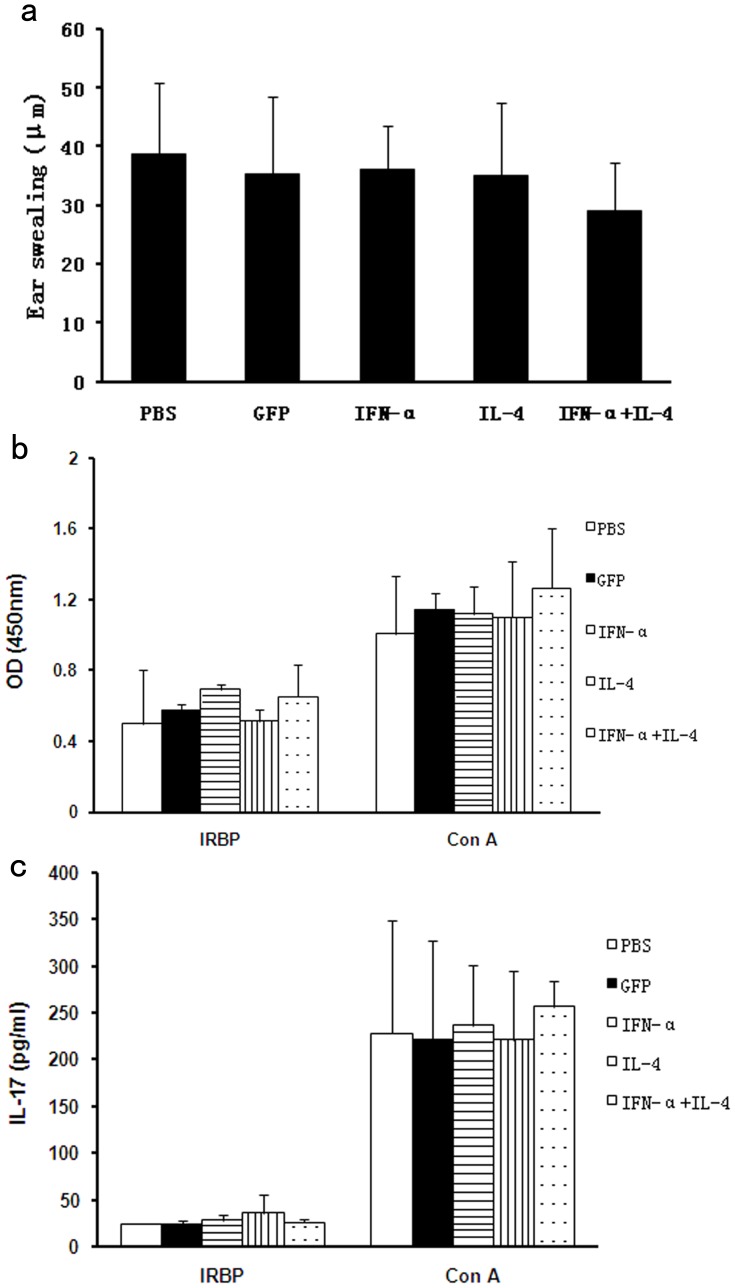
Systemic IRBP-specific immune responses in each group. DTH responses were elicited on day 19 of EAU and evaluated on day 21. Data show no significant difference of ear swelling among all the tested groups (p>0.05) (**a**). IRBP-specific lymphocyte proliferation (**b**) and IL-17 production *in vitro* (**c**) show no significant difference among the five tested groups (P>0.05). Results are presented as mean±standard deviation. 5–6 animals per group were used and each experiment was performed three times.

## Results

### AAV Vectors

The IFN-α or IL-4 gene was driven by the human cytomegalovirus (CMV) promoter and was followed by a BGH poly(A) signal (**Fig. 1**). Titers of recombinant vectors used were 3×10^10^ vg/ml and 7.1×10^10^ vg/ml for AAV2.IFN-α and AAV2.IL-4, respectively. AAV2.GFP was used as a vector control (1×10^11^ vg/ml).

### IFN-α and IL-4 Expression *in vivo* Following Subretinal Injection of AAV Vectors

Mice were subretinally injected with AAV2.IFN-α (1.5×10^7^ vg) alone, AAV2.IL-4 (3.55×10^7^ vg) alone, and AAV2.IFN-α combined with AAV2.IL-4. Two weeks following subretinal injection, the IFN-α and IL-4 level in ocular fluid samples obtained from injected eyes was assayed by ELISA. Data showed that the expression of IFN-α and IL-4 was detectable two weeks following subretinal injection in each group. In the eye receiving an injection of AAV2.IFN-α (1.5×10^7^ vg) alone, the level of IFN-α was 0.128 ng/ml on day 14, and 0.66 ng/ml on day 21 (**Fig. 2a**). The level increased further up to day 42 and started declining on day 90. Following the combined AAV2.IFN-α and AAV2.IL-4 vector injection, expression of IFN-α was observed on day 14, reached a peak on day 42 and remained detectable until three months after injection (**Fig. 2a**). For the expression of IL-4, the eyes injected with the AAV2.IL-4 vector alone and injected with the combined AAV2.IL-4 and AAV2.IFN-α showed a similar expression profile from 14 days to three months after subretinal injection (**Fig. 2b**). For all mice receiving subretinal injection of either AAV2.IFN-α alone, AAV2.IL-4 (3.55×10^7^ vg) alone, or AAV2.IFN-α combined with AAV2.IL-4, IFN-α or IL-4 remained undetectable in undiluted serum and in the contralateral uninjected eyes over time (data not shown).

### The Effect of AAV Vectors on EAU

EAU was successfully induced in B10RIII mice following immunization with 50 µg human IRBP_161–180_ peptide emulsified in CFA as evidenced by conjunctival hyperemia, ciliary injection, corneal edema, posterior synechiae, aqueous flare and cells. The inflammatory signs became apparent between days 8 and 9 following immunization, reached a peak by day 12 and was followed by a gradual regression. None of the control mice receiving CFA alone developed EAU.

To test the effect of AAV vectors on the development of EAU, mice were divided into five groups. Three experimental groups were subretinally injected with either AAV2.IFN-α alone, AAV2.IL-4 alone or AAV2.IFN-α combined with AAV2.IL-4. Another group of mice was injected with AAV2.GFP as a vector control and the fifth group consisted of control mice receiving a subretinal injection with PBS. All groups of mice were immunized with IRBP_161–180_ peptide emulsified in CFA three weeks after subretinal vector or PBS injection.

Clinical signs were monitored after immunization by slit lamp microscopy. In PBS or AAV2.GFP injected eyes, a severe inflammatory reaction including conjunctival hyperemia, ciliary injection, corneal edema, aqueous cells and posterior synechiae was observed (**Figs. 3a, b**). A mild uveitis as manifested by conjunctival hyperemia or ciliary injection was observed in AAV2.IFN-α alone treated, AAV2.IL-4 alone treated, AAV2.IFN-α and AAV2.IL-4 combined treated eyes (**Figs. 3c–e**). Severity of EAU was clinically scored on a scale from 0 to 5. A significantly decreased activity of EAU throughout the course of the disease was observed in the three AAV vector treated groups as compared with the PBS or AAV2.GFP injected controls (**Fig. 3f**). Clinical scoring on day 12 following EAU induction showed that the severity of EAU in AAV2.IL-4 alone injected eyes and AAV2.IL-4 combined with AAV2.IFN-α treated eyes was significantly decreased when compared with PBS and AAV2.GFP injected controls (p<0.0001). Eyes receiving an injection of AAV2.IFN-α alone also showed a significantly decreased inflammation compared with the PBS (p = 0.001) or AAV2.GFP group (p = 0.013). AAV2.IL-4 treatment showed a much more dramatically increased blockade of EAU than AAV2.IFN-α treatment (p = 0.001). Subretinal injection of both AAV2.IL-4 and AAV2.IFN-α showed a stronger inhibitory effect on EAU activity as compared with AAV2.IFN-α administration alone (p<0.0001) and a trend of increased inhibition when compared with AAV2.IL-4 alone, although the difference did not reach statistical significance. (**Fig. 3g**).

Histological analysis showed a severe uveitis in the AAV2.GFP injected and PBS injected control mice as evidenced by massive infiltration of inflammatory cells into the iris, vitreous cavity, throughout all retinal layers and the choroid, intensive retinal vasculitis, destruction of the retinal architecture with severe folding and detachment, as well as photoreceptor damage (**Figs. 4a–d**). However, in AAV2.IFN-α alone treated, AAV2.IL-4 alone treated, AAV2.IFN-α and AAV2.IL-4 combined treated eyes, only scattered infiltration of inflammatory cells into the vitreous body and retina was observed (**Figs. 4e, g, i**). Additionally, the inflammatory changes in the anterior segment of treated eyes were less than those in the AAV2.GFP injected eye and PBS injected eyes (**Figs. 4f, h, j**). Pathological grading showed that the AAV2.IFN-α alone treated eyes showed a significantly decreased uveitis when compared with PBS and AAV2.GFP injected controls (p = 0.01, p = 0.012). AAV2.IL-4 alone injected eyes and AAV2.IL-4 combined with AAV2.IFN-α treated eyes showed more significantly decreased inflammation compared with controls (p<0.0001) than AAV2.IFN-α alone (**Fig. 4k**).

### Effects of Subretinal Injection of AAV Vectors on the IRBP-specific Systemic Immune Response

DTH reactions *in vivo* and lymphocyte responses to IRBP_161–180_ *in vitro* were assayed to evaluate the impact of AAV2 vector subretinal injection on the systemic immune response. Results showed that there was no significant difference in the DTH reactions against IRBP between AAV2.IL-4 injected mice, AAV2.IFN-α injected mice, AAV2.IL-4 combined with AAV2.IFN-α treated mice, AAV2.GFP injected control mice and PBS injected controls (p>0.05) (**Fig. 5** **a**).

Lymphocytes from spleen and lymph nodes were isolated and incubated for 72 hours *in vitro* with IRBP_161–180_ peptide, ConA (positive control), or medium alone (negative control) respectively. The proliferation and IL-17 production of lymphocytes were assayed in lymphocytes obtained from AAV2.IL-4 injected mice, AAV2.IFN-α injected mice, AAV2.IL-4 combined with AAV2.IFN-α treated mice, AAV2.GFP injected control mice and PBS injected controls. The result showed a similar response in proliferation and IL-17 production of lymphocytes incubated with ConA among the five groups of mice. A somewhat lower response in IL-17 production and proliferation of lymphocytes was observed in all the tested groups when exposed to IRBP_161–180_ peptide. There was no difference concerning IRBP-specific lymphocyte proliferation and IL-17 production among all tested groups (p>0.05) (**Fig. 5** **b,c**). Lymphocytes from the tested groups did not show a detectable proliferation and IL-17 production when cultured with medium alone.

## Discussion

In this study, we investigated the effect of ocular gene therapy on the development of uveitis by subretinal injection of recombinant AAV2 vector harboring genes encoding the immunoregulatory cytokines IFN-α and IL-4. Experiments were designed to deliver AAV2.IFN-α alone, AAV2.IL-4 alone, and AAV2.IFN-α combined with AAV2.IL-4, respectively. The results showed an effective expression of the transgene for at least three months without detectable spreading to the systemic circulation or contralateral control eye. Subretinal administration of AAV2.IFN-α alone, AAV2.IL-4 alone, and AAV2.IFN-α combined with AAV2.IL-4 significantly reduced EAU development clinically and histologically. The IFN-α and IL-4 combination vector showed a somewhat more potent therapeutic effect on the development of EAU as compared with the IL-4 vector alone, but the difference did not reach statistical significance. It is possible that experiments using lower amounts of vector would be able to show a synergistic effect. Further studies are needed to address this issue.

Autoreactive effector CD4^+^ T cells play a crucial role in the pathogenesis of autoimmune and autoinflammatory uveitis. Currently these effector cells are divided into the IFN-γ producing Th1, the IL-4 and IL-10 producing Th2, and the IL-17 producing Th17 cells. Abundant evidence is now available to show that both the Th1 and Th17 cells are responsible for uveitis whereas the Th2 cells are regulatory [22], [23], [24]. Most attention for the regulatory capacity of Th2 cells has been directed towards a role for IL-10 [24] and the therapeutic use of IL-4 as mentioned in our study has received little attention so far. Although some studies have reported that the administration of recombinant cytokines showed therapeutic efficacy in clinical uveitis [25], many therapeutic regimens require daily administration due to the short half-life of the cytokine molecules and swift clearance from circulation. Furthermore, extremely high doses are administered systemically to reach a therapeutically active intraocular concentration of the cytokine, and achieving such high systemic levels can potentially result in deleterious side effects [26]. Our previous study showed that the continuous expression of therapeutic cytokine IFN-α by retinal cells after subretinal injection of AAV2 vector containing the IFN-α gene was able to suppress inflammation in mice undergoing EAU [18]. Inflammation could however not to be completely blocked by subretinal injection of AAV2.IFN-α and a combination of immunoregulatory factors might be attempted to get a better protection because of the complex interplay of both pro- and anti-inflammatory molecules involved in EAU development. We therefore designed experiments to explore whether administration of IFN-α, combined with another immunoregulatory cytokine such as IL-4, could achieve a better therapeutic effect on the development of EAU.

IFN-α in the treatment of uveitis has been widely reported in the past several decades. According to previous reports, systemic administration of IFN-α in patients with Behcet’s disease and other immune-related disorders could help to upregulate Tregs and inhibit IL-17-expressing cells [27], [28], [29]. Additionally, other reports have shown that circulating levels of TNF-α and TNF-α2R in patients with Behcet’s disease were decreased after IFN-α treatment [30]. Another cytokine, IL-4, has been successfully used in autoimmune animal models in the past few years [8], [9], [31]. Delivery of IL-4, basically, helps to excite a Th2 response and may be beneficial in those autoimmune diseases, whose development depends on the polarization of Th1 cells. Recently, IL-4 has been reported to be able to induce the development of CD25+CD4 T cells with regulatory capacity and that these cells play a prominent role in the development of Ag-specific CD25+CD4 Tregs in vivo [32], which has been thought protective to EAU. Local delivery of IL-4 has been shown to increase the recruitment of Treg cells by increasing the synthesis of chemo-attractant cytokines in inflamed CNS areas [9]. Generally, contribution to upregulate the local Treg population by IL-4 *in vivo* could be another arm of the anti-inflammatory pathways. However, the expression of inflammatory cytokines, chemokines as well as the target cell in ocular lesions remains to be clarified and the exact mechanisms through which IFN-α and IL-4 function in the treatment of EAU need to be investigated further in future studies. Our current experiments suggest that the inhibitory effect on the development of uveitis is mediated via a local and not due to a systemic effect, since we were not able to detect an effect of local cytokine vector delivery on the DTH reaction against IRBP nor on the IRBP specific lymphocyte response in mice undergoing IRBP induced EAU. Consistent with these results, a similar phenomenon in this study and previous ones is that AAV2-mediated transgene expression was restricted to the injected eye without detectable spreading in the blood. The fact that the systemic immune response is not affected by retinal gene delivery may avoid the possibility of systemic immunosuppressive side effects.

In conclusion, we have now confirmed that AAV2-mediated subretinal delivery of IFN-α and IL-4 could effectively attenuate EAU in mice in the absence of systemic immunosuppression. A combined delivery of IL-4 and IFN-α did not result in a synergistic effect. A further understanding of the exact mechanisms by which the released IFN-α and IL-4 exactly inhibit EAU will contribute to the development of efficient and safe retinal gene therapy in uveitis.
